# Presentation and Management of Pulmonary Sequestration With an Aneurysmal Aberrant Pulmonary Artery

**DOI:** 10.7759/cureus.60225

**Published:** 2024-05-13

**Authors:** Chadi C Nahal, Tyler Lackland, Hannah Lowe, Joseph Platz

**Affiliations:** 1 Department of Surgery, Saint Louis University School of Medicine, St. Louis, USA

**Keywords:** wedge resection, thoracoscopy, endovascular embolization, aneurysm, pulmonary sequestration

## Abstract

Bronchopulmonary sequestration presents rarely in adults and less frequently with an aneurysmal aberrant feeding artery. Treatment of bronchopulmonary sequestration generally involves lung resection with vascular ligation; however, aneurysmal disease increases the risk of intra- and postoperative hemorrhage and often necessitates more extensive surgery for vascular control. A 39-year-old female patient with a history of prior abdominal surgery presented with sudden onset epigastric and back pain. Computed tomography demonstrated an aneurysmal aberrant pulmonary artery originating from the abdominal aorta, adjacent to the celiac artery, supplying an intralobar pulmonary sequestration in the inferior right lower lung lobe. She also had evidence of cholelithiasis, with confusing symptom correlation. She was treated with a minimally invasive hybrid approach, which involved endovascular arterial embolization prior to delayed thoracoscopic lung resection. This is a safe and effective approach that reduces the risk of intraoperative bleeding while safely achieving vascular control proximal to the aneurysmal disease.

## Introduction

Bronchopulmonary sequestration (BPS) is characterized by extra-anatomic lung tissue that does not contribute to respiratory function [1]. It receives blood supply from the systemic circulation, primarily the thoracic (87.4%) and, less commonly the abdominal (10.5%) aorta [2]. It is believed to be secondary to deviant lung tissue during embryological foregut migration, typically presenting in the left lower lobe, and is more frequently intralobar than extralobar. The diagnosis of an intralobar sequestration occurs in individuals younger than 20 years old approximately 60% of the time; it is significantly rarer to find BPS in patients older than 50 years [3].

Diagnosis of BPS in adults is often incidental but is traditionally evaluated with computed tomography (CT) or magnetic resonance imaging (MRI) [4]. Chronically persistent and/or recurrent episodes of pneumonia and hemoptysis can be common manifestations [3]. In a retrospective review of 2,625 cases of BPS, 67.6% of patient presentations had cough, 38.95% fever, and 27.67% hemoptysis [5]. Even less common than BPS alone is the presence of aneurysmal disease in the aberrant feeding pulmonary vessel. Although the etiology is unknown, it has been postulated that aneurysmal changes can be due to thin-walled pulmonary vasculature being placed under systemic pressure, leading to accelerated atherosclerosis and vessel wall degeneration [6].

We present the case of a 39-year-old female with a history of previous abdominal surgery with pulmonary sequestration and an aneurysmal feeding pulmonary artery.

This article was previously presented at the 2024 MO ACS Annual Meeting on April 21, 2024.

## Case presentation

A 39-year-old female with a history of obesity, hypertension, depression, sleep apnea, and early-stage ovarian malignancy treated with oophorectomy presented to an outside hospital for sudden-onset epigastric and back pain. CT demonstrated a dilated (2.2 cm) and partially thrombosed anomalous pulmonary artery communicating between the inferior right lower lobe and the abdominal aorta. Additionally, CT showed cholelithiasis without cholecystitis. She was transferred to our hospital for a higher level of care. On arrival, she continued to have abdominal and back pain. She was afebrile with a systolic blood pressure of 170 mmHg, a heart rate of 70 beats per minute, and a Hgb of 11.5 gm/dL.

**Figure 1:**
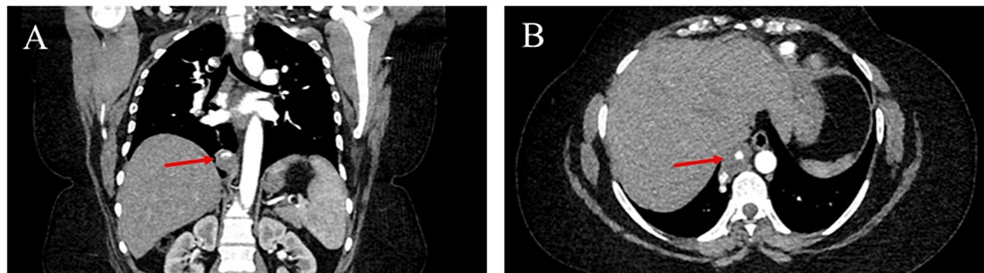
CT angiography of the pulmonary sequestration Computed tomographic angiography demonstrates pulmonary sequestration with an aberrant aneurysmal arterial supply in the (A) sagittal and (B) coronal views.

Computed tomographic angiography (CTA) demonstrated pulmonary sequestration in the inferior right lower lobe with arterial supply from an aberrant artery originating from the abdominal aorta, just adjacent to the celiac artery. The aberrant artery was noted to not only be of small caliber in the abdomen, with a tortuous retroperitoneal course, but also to have significant fusiform aneurysmal dilation as the vessel entered the thoracic cavity at the right cardiophrenic angle (Figure 1).

**Figure 2:**
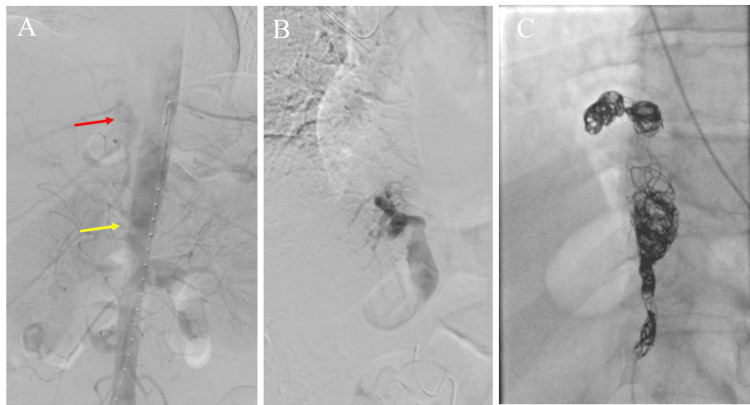
Angiogram of aberrant pulmonary artery The angiogram demonstrates an aberrant pulmonary artery (red arrow) originating adjacent to the celiac axis (yellow arrow) (A) with aneurysmal dilation before (B) and after (C) coil embolization.

Resection of the sequestered lung was warranted but given the appearance of the intrathoracic portion of the feeding vessel, without healthy proximal vascular tissue, primary intrathoracic ligation was deemed unsafe and high risk. Similarly, per discussions with the vascular surgery team, intraabdominal surgical ligation was considered risky given the tortuous course, proximity to celiac vessels, and previous open abdominal surgery. Therefore, endovascular embolization was pursued. Multiple coils were deployed within the aneurysm sac and just distal to the arterial origin. Post-deployment angiogram demonstrated successful embolization with a complete lack of intraarterial flow (Figure 2).

**Figure 3:**
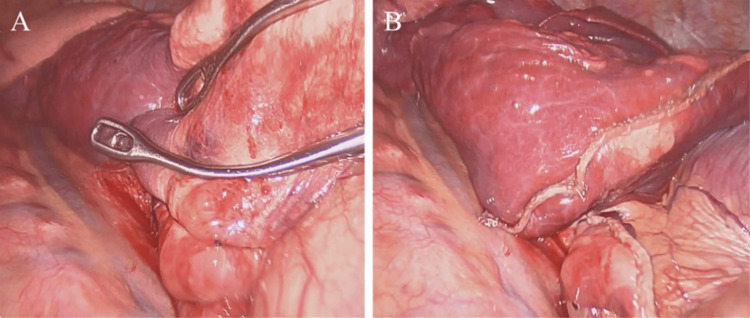
Thorascopic wedge resection of pulmonary sequestration Intraoperative imaging shows the pulmonary sequestration, anomalous aneurysmal vessel, (A) and the residual tissue following stapled resection (B).

Two days later, the patient underwent video-assisted thoracoscopic surgical (VATS) wedge resection of the sequestration portion of the right lower lobe (Figure 3). The anomalous pulmonary vessel was identified after appropriate superior lung retraction, entering the pleural cavity in the cardiophrenic angle, adjacent to the inferior pulmonary ligament. The entire intrathoracic vessel was confirmed to be aneurysmal but was also felt to be completely thrombosed by the time of surgery. The anomalous artery was circumferentially dissected free from the diaphragm to its lung insertion and ligated twice with 0 silk and Ti-Knot. This was followed by stapled wedge resection of the pulmonary sequestration and stapled division of the aberrant vessel. The patient was extubated and taken to the recovery room in stable condition.

The patient did quite well postoperatively with symptom resolution and without complications. Her chest tube was pulled on postoperative day 1, and she was discharged in good condition the following day.

## Discussion

Our patient with pulmonary sequestration presented with non-specific symptoms similar to those previously reported, specifically upper back and abdominal pain [7]. Uniquely, this case of pulmonary sequestration included aneurysmal disease of the aberrant pulmonary artery. The etiology is unclear, but the patient’s medical history of obesity and hypertension may have contributed to aneurysm formation over time.

Some have argued for the treatment of pulmonary sequestration without surgery, using only endovascular approaches to mitigate bleeding risk [8]; however, the generally recommended treatment for BPS is the removal of the aberrant lung tissue in both symptomatic and asymptomatic patients [3]. This avoids recurrent infection in non-functional lung tissue. Surgical lung resection generally includes vascular ligation of the extra-anatomic vasculature from the chest. With the presence of an aneurysmal feeding vessel, ligation may be required at the vessel’s origin or at least proximal to the aneurysmal dilation; this may necessitate patching of the aorta via thoracotomy or thoracoscopy [9,10]. Aneurysmal disease complicates the surgical treatment of pulmonary sequestration, as it increases the risk of intra-operative bleeding, makes vascular control more difficult, and is associated with aneurysmal recurrence following resection [6]. In this case, we performed endovascular embolization before VATS wedge resection of the BPS, avoiding the risk of intrathoracic bleeding during lung resection, as well as the need for a separate abdominal surgery. An alternative approach involves thoracic endovascular stent exclusion of the pulmonary artery prior to lobectomy, which involves placement of an aortic stent graft over the aberrant arterial origin to exclude arterial flow from the aorta [11-13]. This option, however, is generally used with an aberrant artery of thoracic origin and would have led to occlusion of the celiac and possibly superior mesenteric arteries in our patient. Endovascular treatment before surgical resection minimizes the bleeding risk of ligating an aneurysmal vessel and avoids the need for surgical ligation in a separate operative field, particularly when the aberrant artery originates in the abdomen. The drawbacks of the hybrid approach include a prolonged hospital stay for two separate procedures and the possibility of incomplete vascular control when compared to surgical ligation [1,6].

In our case, endovascular embolization of the aberrant aneurysmal artery before wedge resection avoided the need for open abdominal vascular ligation, along with possible injury to the celiac artery injury and intraoperative bleeding during thoracoscopic lung resection. Particularly when managing an aberrant feeding vessel of pulmonary sequestration that is difficult to safely access or is of poor tissue quality, we suggest that endovascular occlusion prior to definitive lung resection is an excellent option.

## Conclusions

When managing pulmonary sequestrations, a thorough assessment of patients’ vascular anatomy and medical and surgical history is necessary. For our patient, the proximity of the aberrant artery to the celiac axis and adhesions related to previous surgeries would have increased the risk of iatrogenic injury to intra-abdominal organs and vascular structures if she were to undergo intra-abdominal ligation of the aberrant artery. Therefore, endovascular embolization before thoracoscopic wedge resection and suture ligation of the aberrant artery allowed for the minimization of these risks and permitted complete occlusion of the vasculature before lung resection. Because aneurysmal aberrant pulmonary arteries have a higher risk of bleeding during resection, preoperative endovascular embolization can be an effective and safe method for decreasing intraoperative bleeding during the resection of pulmonary sequestrations.
